# Osteogenic Differentiation of Mesenchymal Stem Cells via Curcumin-Containing Nanoscaffolds

**DOI:** 10.1155/2021/1520052

**Published:** 2021-07-17

**Authors:** Khadijeh Khezri, Solmaz Maleki Dizaj, Yalda Rahbar Saadat, Simin Sharifi, Shahriar Shahi, Elham Ahmadian, Aziz Eftekhari, Elaheh Dalir Abdolahinia, Farzaneh Lotfipour

**Affiliations:** ^1^Deputy of Food and Drug Administration, Urmia University of Medical Sciences, Urmia, Iran; ^2^Dental and Periodontal Research Center, Tabriz University of Medical Sciences, Tabriz, Iran; ^3^Kidney Research Center, Tabriz University of Medical Sciences, Tabriz, Iran; ^4^Faculty of Dentistry, Tabriz University of Medical Sciences, Tabriz, Iran; ^5^Pharmacology and Toxicology Department, Maragheh University of Medical Sciences, Maragheh, Iran; ^6^Research Center for Pharmaceutical Nanotechnology, Biomedicine Institute, Tabriz University of Medical Sciences, Tabriz, Iran; ^7^Food and Drug Safety Research Center, Tabriz University of Medical Sciences, Tabriz, Iran; ^8^Faculty of Pharmacy, Tabriz University of Medical Sciences, Tabriz, Iran

## Abstract

The diverse pleiotropic pharmacological effects of curcumin nanoformulations have turned it into an attractive natural compound in different health-related problems. A great body of evidence has shown the impact of curcumin and its nanoformulations on the differentiation of stem cells. The current review highlights cellular and molecular mechanisms connected with the osteogenic differentiation of mesenchymal stem cells (MSCs) in the scaffolds benefiting from the presence of nanocurcumin pointing toward the role of inhibitory or stimulant signal transduction pathways in detail. Moreover, the effects of different concentrations as well as the structural modifications of curcumin on the differentiation of MSCs have been addressed.

## 1. Introduction

Studies have shown that a range of therapeutic effects of various herbal medicines can be identified [1–4]. Curcumin (diferuloylmethane) as a bioactive hydrophobic polyphenolic ingredient is obtained from *Curcuma longa* (turmeric) rhizome. Recently, it received significant attentions as a medicinal plant due to its unique therapeutic benefits including antipathogenic, antioxidant, anti-inflammation, antiangiogenic, anticancer, and antidiabetic effects, as treatment for central nervous system and cardiovascular disorders, in skin diseases and the cosmetic industry, lung diseases, antiatherosclerotic, ophthalmic, cystic fibrosis and osteoporosis, beta-thalassemia, *etc.* [5–8]. Also, it was reported as a valuable material in nutritional supplements due to the pleiotropic pharmacological properties [9]. Despite its multitarget biological effects, there are limitations to its clinical use due to its low absorption, bioavailability, and short half-life. Recently, several nanoparticles such as solid lipid nanoparticles, nanostructured lipid carriers, liposomes, micelles, nanogels, and magnetic nanoparticles are developed as novel and potential therapeutic strategies to improve therapeutic effects of various drugs such as curcumin [5–11].

Various investigations have demonstrated that novel systems of curcumin and stem cell differentiation have wonderful therapeutic potential against various bone-related diseases [12]. An enhancement in adipocyte number in obesity can be the result of extreme differentiation of mesenchymal stem cells (MSC) to cell lineages of adipose. Osteoclasts and osteoblasts are recently recognized as the most main bone-resorbing and bone formation cells, respectively, during bone resorption. It is reported that excessive bone resorption leads to metabolic bone disorders such as bone loss and osteoporosis [13, 14]. A number of studies have shown that various formulations of curcumin can be used as a therapeutic agent for the treatment of obesity and osteoporosis. It has a capability to the differentiation of MSCs into the mesodermal lineage such as chondrocytes, osteoblasts, and adipocytes [2, 15]. Recent investigations revealed that MSCs can be used as a promising and effective therapeutic agent in bone tissue engineering and regenerative medicine [8, 9, 12].

Herein, we highlighted the cellular and molecular mechanisms connected with the osteogenic differentiation of mesenchymal stem cells (MSCs) in the scaffolds benefiting from the presence of nanocurcumin pointing toward the role of inhibitory or stimulant signal transduction pathways in detail.

## 2. The Osteogenic Differentiation Activity of Curcumin

In recent years, the osteogenic differentiation activity of curcumin has been reported due to its unique therapeutic effects in the treatment of various bone diseases such as protecting against ovariectomy-induced bone loss, increasing bone mineral density, preventing osteoporosis and arthritis, and improving bone microarchitecture [16–18]. The results of these studies revealed that curcumin formulations have high therapeutic capacity to induce bone remodeling by the suppression and/or induction of osteocyte differentiation.

Results of a study demonstrated that an ethanolic solution containing curcumin can have a positive effect on osteoblast differentiation of precursor cells. Also, these investigations showed that curcumin as a direct-acting agent applied its osteoblast differentiation effects through enhancing the expression of bone-associated gene markers including osterix, runt-related transcription factor (Runx2), and bone morphogenetic protein 2 (BMP-2) in MSCs *in vitro* [19]. They showed that the use of curcumin additives can increase activity of markers of mature osteoblasts such as alkaline phosphatase (ALP), mineralized nodules, and osteocalcin (OCN) expression [20]. In this regard, studies on curcumin formulations indicated that it can be used to improve its transdifferentiation. The experimental results showed that curcumin considerably improved hLMP-3 osteogenic function and cell transdifferentiation action through the enrichment of differentiation medium with curcumin [21].

The effects of curcumin on several signaling pathways were studied, such as Akt/GSK3*β*, Wnt/TCF and Wnt/*β*-catenin, 1/Nrf2/HO-1, and ER stress. The protocols of curcumin use in cell culture are briefly described in Table 1.

## 3. Akt/GSK3*β* Signaling Pathway

In recent studies, it was revealed that the antioxidant activity of curcumin has a main effect in osteoblast differentiation [26]. These studies showed that reactive oxygen species (ROS) and oxidative stress inhibit the osteoblastic differentiation of MSCs [27]. In accordance with this property, the importance of the antioxidant activity of curcumin was highlighted for protection of stem cells from oxidative stress damages, osteoblast apoptosis, and osteoporosis [28]. Increasing evidences show a relationship between overproduction of ROS and mitochondrial dysfunction. Recently, curcumin supplements are introduced as decreased agents of mitochondrial oxidative damage and can be considered as a promising therapeutic approach for the improvement of physiological functions of mitochondria during treatment with curcumin formulations. Curcumin is also known as a promising natural preservative agent for the mitochondrial redox status through enhancing the amounts of phosphorylated protein kinase B (Akt) and phosphorylated glycogen synthase kinase-3*β* (GSK3*β*). Results of this study revealed that the activation of Akt-GSK3*β* signaling by curcumin can improve oxidative stress-induced apoptosis in osteoblasts through protecting mitochondrial capacities [22].

It also showed that curcumin can be utilized as a potential protective mechanism against mitochondrial dysfunction in key metabolic organs such as the kidney, liver, and brain. It appears that signaling pathways such as PI3K/Akt may have an interaction with this protective mechanism [29, 30].

## 4. Wnt/TCF and Wnt/*β*-Catenin Pathways

Wnt/*β*-catenin signaling pathway has a vital effect in bone formation, repair, and modulation of osteogenic differentiation. This pathway can be inhibited through dickkopf-1 (Dkk-1). Dkk-1 is characterized as an extracellular Wnt inhibitor. This study revealed that osteogenic differentiation capacity of cells decreased by Dkk-1 inhibition [31]. A cytoplasmic cascade suppressed the activity of GSK3*β* signaling pathway through attaching ligands to the Wnt receptor. It has been reported that GSK3*β* can induce a special signaling pathway for the degradation of *β*-catenin through GSK-3*β*–*β*-catenin interaction. Previous results have shown that inactivation of the GSK3*β* pathway inhibits the proteasomal phosphorylation and degradation of the cotranscription factor *β*-catenin.

By transferring it to the nucleus and attaching to T-cell factor (TCF), the Wnt/TCF pathway is activated and osteogenic differentiation is increased [32]. Recently, curcumin formulations are emerging as approaches to develop and improve the expression of Wnt receptors and coreceptors such as Fz2 and LRP 5/6, respectively [33]. Suppressing the degradation activity of H_2_O_2_ on *β*-catenin and inducing ROS scavenger enzymes such as HO-1 and its critical proteins including cyclin D1 and C-myc are known as mechanisms of the antioxidant activity of curcumin for support of the Wnt/*β*-catenin pathway [31]. Activity of the *β*-catenin-mediated oxidative stress can inhibit the Wnt/TCF pathway by expression of forkhead box O (FoxO) transcription instead of TCF. As previously mentioned, curcumin actives the Wnt/TCF pathway by downregulation of oxidative stress, so curcumin can inhibit Foxo1–*β*-catenin-mediated oxidative stress pathway. Also, use of curcumin supplements can increase the expression of cyclin D1 and C-myc proteins through amplifying the Wnt/*β*-catenin pathway [23].

## 5. 1/Nrf2/HO-1 Pathway

Curcumin can boost the antioxidant system in MSCs through the upregulation of heme-oxygenase-1 (HO-1) and stimulation of kelch-like ECH-related protein 1 (Keap1)/Nrf2 (nuclear factor erythroid 2-related factor 2)/HO-1 cascade which also can diminish the oxidative hazard, enhance cellular resistance to oxidative damage, and promote osteoblast differentiation [34]. The hydrogen binding of distinct amino acids (Ile458 and Arg441) in the structure of the Nrf2 proteins with curcumin results in the direct effect of this agent on receptors [35]. The activation of HO as an enzyme catalyzer of heme degradation upon HO-1 expression can result in the differentiation of MSCs to osteocytes [36]. Scanty experiments have shown upregulation of HO-1 in cancerous cells, hepatocytes, fibroblasts, and cardiac cells subsequent to curcumin treatment in which the activation of MAPK signaling pathway by curcumin is in charge for the expression of HO-1 [28, 37].

Curcumin loaded into polylactic glycolic acid (PLGA) microspheres has been utilized to prohibit ROS generation in MSCs and improve osteogenesis. In this study, curcumin-encompassed microsphere and MSC incorporation into a collagen/hydroxyapatite composite scaffold was performed. Curcumin was liberated persistently from the scaffold for the next 30 days and plunged the production of H_2_O_2_ in the diabetic serum. Moreover, the downregulation of nicotinamide adenine dinucleotide phosphate oxidase 4 (NOX4) along with surged expression of manganese-dependent superoxide dismutase (MnSOD) gene enhanced mitochondrial function in relation to curcumin. The Keap1/Nrf2/HO-1 pathway was also activated by curcumin through upregulation of the total HO-1 and Nrf2 genes and decreases Keap1 expression which in turn decreases ROS formation and enhances the osteogenic differentiation of MSCs as evidenced by the elevated expression of osteogenic markers such as Runx2, OPN, and OCN genes [38].

## 6. ER Stress Pathway

Curcumin exerts the same effects as BMP2 (a cytokine in osteoblast differentiation) on MSC differentiation into osteoblasts. It stimulates the Smad 1/5/8 signaling pathway which in turn activates Runx2 and osteogenic marker expression. The Smad-mediated Runx2 activation leads to the slight mediation of reticulum endoplasmic (ER) stress through the stimulators of preliminary unfolded protein response (UPR) such as inositol-requiring enzyme 1 (IRE-1), protein kinase R- (PKR-) like endoplasmic reticulum kinase (PERK), and activating transcription factor 6 (ATF6). Also, curcumin and BNP2-associated cascades induce the upregulation of ER stress marker genes such as CCAAT/enhancer binding protein (C/EBP) homologous protein (CHOP), immunoglobulin binding protein (BiP), ER degradation-enhancing-*α*-mannidose-like protein (EDEM), and ATF4. Moreover, the upregulation of OCN and differentiation of MSCs to osteocytes are observed upon overexpression of ATF6 [20].

## 7. Modifications of Curcumin

Molecular modifications/conjugations of curcumin may extensively enhance its activity and bioavailability as well as the type of differentiation. For instance, the deacetylated curcumin exerted lower inhibitory potential on the adipogenic differentiation of 3T3-L1 in comparison to the acetylated form. It has been suggested that the hydrogen bond donor in the free phenol group of the deacetylated form can interfere with adipocyte differentiation [39]. Gupta and colleagues stated that molecular modifications of curcumin interact with the binding sites or receptors on the MSC membrane [40]. Curcumin interactions with cell membrane proteins result in MSC differentiation into each of the three mesodermal lineages through the activation or inhibition of the aforementioned transduction signaling pathways. Evidence from literature confirmed that the curcumin structure (free or in association with other biomaterials) exerted various differentiation potentials since its integration into materials alters cell interaction in different aspects. The interaction of free curcumin with the binding sites of the cellular membrane is quite rapid and easy in comparison with bound curcumin. [41]. Cultivation of MSCs on curcumin-entrapped silk hydrogel films leads to the adipogenic differentiation, whereas the equal amounts of curcumin utilized in solution on culturing MSCs resulted in the prohibition of adipogenic differentiation and reduced the number of lipids containing cells. Moreover, structural analysis of silk-functionalized curcumin indicated the interaction of curcumin hydrophobic molecules with hydrophobic beta-sheet domains of silk structure, which in turn results in induction of the alternations in the silk secondary structure from random coil to beta sheet [41]. In another study, applying curcumin-34-dichloro phenyl pyrazole (CDPP) significantly enhanced adipogenesis inhibition *in vitro* and *in vivo* gastrointestinal stability and bioavailability in comparison to the free curcumin [42]. Heo et al. performed a study to investigate the effects of gold nanoparticles functionalized with cyclodextrin curcumin complexes on the osteoclast differentiation inhibition. Using an ovariectomy- (OVX-) induced osteoporosis model, they demonstrated that CUR-*β*-cyclodextrin- (CD-) conjugated gold nanoparticles (CUR-CGNPs) efficiently improved bone density and prevented bone loss and consequently could be considered as novel therapeutic alternatives in osteoporosis prevention and treatment [43].

The osteoclast-related bone resorption, inflammatory response, and the occurrence of apoptosis were investigated in an experimental model of periodontitis after the intake of curcumin and its chemically modified type (CMC2.24). It was found that the number of osteoclasts and alveolar bone resorption was decreased in CMC2.24 received mice. Moreover, the number of apoptotic cells in the gingival tissues as well as the amount of osteocytes was plummeted in curcumin-administrated animals while CMC2.24 exhibited no noticeable effects in this context [44].

Curcumin has been modified to increase its effectiveness, and limited research has been done on its limitations. Investigations on the bioactivities of curcumin conjugates relative to the free curcumin molecule have been previously carried out. Some studies displayed revealed that curcumin conjugates with glucuronides exhibited poor antiproliferative and antioxidant properties compared to curcumin in its unconjugated form [45, 46].

Nanocarriers have been utilized for delivery of curcumin to circumvent the substance's bioavailability that seriously limits its application for therapeutic ends in patients [47, 48]. It has been found that the nanocarrier's size can change the effect of their haul and may present some cytotoxicity or immune response depending on the chemical/physical properties [49].

## 8. Curcumin-Containing Scaffolds

Every day, a large number of surgeries are performed to replace or repair damaged tissue in the world. The field of tissue engineering is being developed with the aim of regenerating damaged tissues or guiding the growth of new tissue. Numerous scaffolds made from a variety of biomaterials and made using a variety of fabrication methods have been used in an effort to regenerate various tissues and organs.

Curcumin has been used to treat various damaged tissues, especially wound injuries. There are several types of formulations containing curcumin, among which nanoformulations are of particular importance in restorative medicine and tissue engineering. The design of drug delivery systems for controlled/targeted delivery of curcumin to tissues and organs is also of great importance. Among several techniques described in the literature, electrospinning has gained important attention due to capability of forming various nanofibrous morphologies owing to its functionality, simplicity, and flexibility. The applied voltage and needle to collector distance flow rate, as well as solution parameters such as surface tension, viscosity, and electrical conductivity of the solution, are among variables of electrospinning process which in turn control the nanofibers morphology. Identifying conditions of forming fibers with a minimum diameter is considered as a final aim of electrospinning procedure optimization.

**Figure 1:**
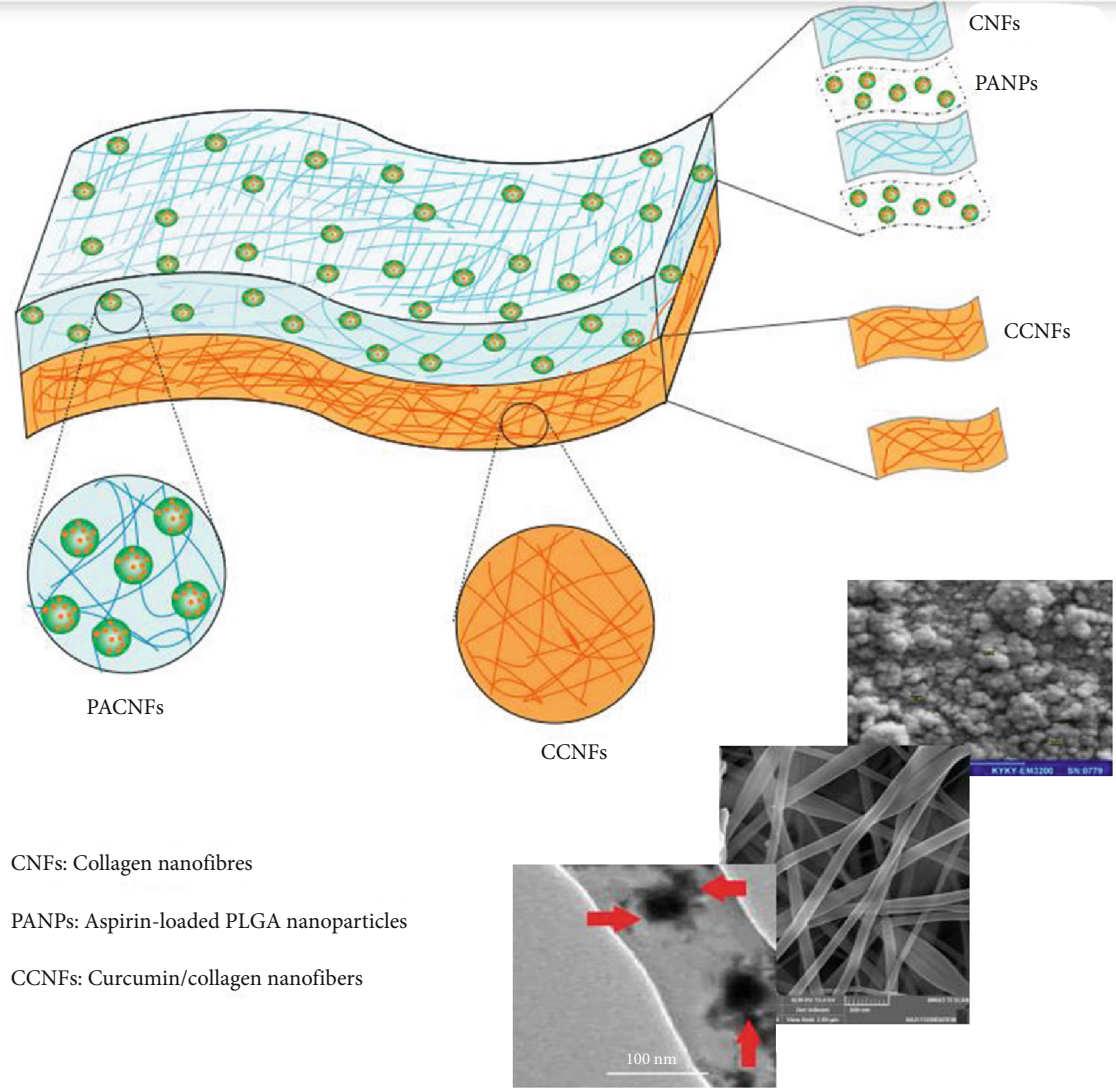
Combination of curcumin and aspirin in asymmetric multifunctional GBR nanofibrous scaffold.

In a study conducted by our group, curcumin in combination with aspirin was utilized to develop an asymmetric multifunctional guided bone regeneration (GBR) nanofibrous scaffold by means of electrospinning procedure with the optimum settings of 20 kV voltage, distance of 10 cm between a collector, and a capillary tube with 1.5 ml/h flow rate. The physicochemical findings demonstrated a mean size of 84.06 nm curcumin nanofibers and a randomly electrospun fibers on the collector which exerted a high resemblance to the ECM construction. Besides, the results of the study revealed the antibacterial activity of the scaffold against *Staphylococcus aureus*, *Escherichia coli*, and *Enterococcus faecalis*. According to our cellular results, curcumin could substantially influence the differentiation of dental pulp stem cells (DPSCs) proved by enhanced expression of osteogenic markers including Runx-2 and OCN 21, days after treatment. Also, it was observed that the level of osteogenic proteins was higher posttranslations, which indicates the potential role of curcumin in induction of DPSC differentiation at both translational and transcriptional stages. In order to evaluate the bone regeneration performance of the fabricated scaffold in the alveolar bone defect model, six adult mongrel dogs were exploited. Defects (8 mm in diameter and 4 mm deep) were performed on both sides of the models' jaw, of which one side was utilized as the test side for assessing the suitability of the fabricated scaffold and the other covered with Regen collagen (a commercial membrane) as the control side. The findings of this *in vivo* study demonstrated complete repair of bone after 28 days; however, the side covered by the commercial membrane was unable to fill the defect. The pathology specimen showed no inflammation besides the membranes degraded fully. New bone formation was seen in the histological test of the prepared membrane side, but on the other side (commercial membrane side), defect edges were sharp and intact. Cellular experiment results demonstrated osteocondutive properties of the curcumin-containing nanofibrous scaffolds; moreover, it can function as a physical barrier for the GBR technique. Furthermore, the wound healing effects of curcumin lead to the formation of the soft tissue above the new bone. The findings of the antimicrobial tests revealed that the formed scaffolds can be utilized as a local antimicrobial delivery structure for inhibiting infections in the surgical area [5].  Figure 1 shows the prepared curcumin and aspirin containing asymmetric multifunctional GBR nanofibrous scaffold by our group.

In a study by Jain et al., electrospinning method was successfully employed for the preparation of curcumin-loaded poly (*ε*-caprolactone) (PCL) nanofibers. The particle size of nanofibers including neat fibers and fibers with 1 wt% and 5 wt% curcumin was in the range 840 ± 130 nm, 827 ± 129 nm, and 680 ± 110 nm, respectively. The results of 1H nuclear magnetic resonance and Fourier transformation infrared spectroscopy analysis showed that curcumin was well encapsulated within the nanofibers. The obtained results of release test in aqueous medium exhibited a biphasic drug release profile consisting of an 18% initial release in 3 d and 60% controlled drug release in 9 d. In this study, assessment of encapsulation efficiency of curcumin on MC3T3-E1 preosteoblasts by evaluating the expression of staining of mineral deposits and ALP using alizarin red stain showed that curcumin nanofibers significantly modulated the expression of osteogenic markers in cell proliferation (osteogenesis) through increasing the percentage of curcumin encapsulation in nanofibers (5% curcumin) compared with the neat polymer and 1% curcumin. Also, the results of FTIR analysis affirmed the formation of mineral sediment. Results of sustained curcumin release profile of polymer scaffolds showed that these nanoparticles can be used as a novel therapeutic approach for treatment of bone tissue regeneration disorders [50].

It has been reported that incorporation of 3D printing (3DP) of calcium phosphate (CaP) porous scaffolds with nanocarriers such as curcumin-loaded liposomes increases its bioavailability. New and promising strategies in engineering and fabricating 3DP scaffolds (such as special shape and interconnected porosity) have led to the design of implants with the ability to regrow new tissue. The results of cytotoxicity assays of 3DP scaffold-loaded curcumin liposome formulation on both human fetal osteoblast cells (hFOB) and human osteosarcoma (MG-63) cell lines (after 11 days of incubation) revealed that these nanoparticles have significant toxicity against MG-63 cell line compared to hFOB. Also, the cell viability, proliferation, and differentiation of osteoblast cells were significantly increased using 3DP scaffold-loaded curcumin liposome formulation. Using these promising therapeutic approaches might open new avenues for treatment of bone defects after tumor resection [51].

**Figure 2:**
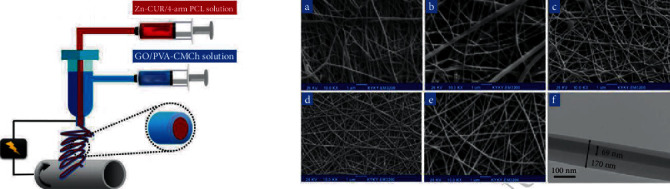
The scaffolds of coaxial electrospun nanofibers using GO and Zn-CUR complex for bone regeneration [52] (adopted with permission).

Recently, a new approach for bone tissue engineering suggested the development of bone substitute substances using 3D bioactive composite scaffolds of biocompatible polymers. In another study, Sedghi et al. designed scaffolds of coaxial electrospun nanofibers using graphene oxide (GO) and Zn-curcumin complex (Zn-CUR) and evaluated the potential of these scaffolds for bone regeneration (Figure 2). The TEM, SEM, and FT-IR techniques were used to determine the physicochemical properties of the electrospun nanofiber scaffolds. The obtained results of TEM and SEM images confirmed that nanofibers had nanosize (153 nm) and defect-free uniform coaxial forms. In this study, bioactivity and cytocompatibility of the composite nanofibers were assessed by ALP activity, alizarin red S (ARS) staining (as a bone-staining agent), and MTT assay. Morphological characterization of the MTT cell viability assay showed that Zn-curcumin nanofibers significantly increased cellular adhesion, diffusion, and proliferation than drug-free nanofibers. It reported that scaffolds containing Zn-curcumin nanofibers potentiate the osteogenic performance, ALP function, and the generation of matrix mineralization. Due to the antibacterial properties of zinc-curcumin-loaded coaxial nanofibers, it can also be used as an effective therapeutic agent for the treatment postoperative infections. Conclusively, the findings of this study suggested that the composite scaffolds containing Zn-CUR nanofibers have the high capacity to develop bone tissue engineering therapies [52].

## 9. Summary and Future Perspectives

In the present review article, the main aim was to focus on curcumin capacities in the osteogenic differentiation of MSCs and evaluation of its various molecular mechanisms. Curcumin-containing nanoscaffolds were also evaluated as a new type of drug delivery system to improve curcumin's biological activities as well as scaffold's functionality. A review of recent studies demonstrated that AMPK regulation and activating the Wnt signaling pathway are main mechanisms of curcumin for suppression of MSCs from adipogenic differentiation. The findings of this study revealed that curcumin formulations are also able to decrease the expression of molecular mechanisms associated with MSCs' proliferation, adipogenic markers, and Fas expression by binding to PPAR*γ* receptors. In these studies, ER stress, Wnt/*β*-catenin, Akt/GSK3*β*, and Keap1/Nrf2/HO-1 signaling pathways were introduced as potential mechanisms of curcumin for the induction of osteoblastic differentiation of MSCs. These findings demonstrated that curcumin can prevent osteoclast and osteoblast differentiation through the BMP/Smad and JNK/Bax mechanisms. Also, these results showed that curcumin has a more potent inhibitory effect against osteoclast differentiation through inhibition mechanisms of RANKL/RANK and NF-*κ*B and activation of Wnt/*β*-catenin pathways. Curcumin could increase chondrogenic differentiation activity of MSCs through an anti-inflammatory mechanism, and it could also reduce by its effect on inhibiting actin reorganization and stimulating apoptosis. It was reported that the delivery system type of curcumin (loaded or free), its dose, and form can greatly affect its activity on mesodermal lineage differentiation. Therefore, formulations with novel drug delivery systems can improve its functional and biological activities. With attention to the potential capacities of curcumin on the osteogenic differentiation of cells, it could be developed as a promising and effective therapeutic agent for a variety of clinical applications such as the therapy for osteoporosis disorders. The field of tissue engineering needs to be developed with the aim of regenerating damaged tissues or guiding the growth of new tissue using a variety of biomaterials like curcumin.

## Figures and Tables

**Table 1:** The protocols of curcumin use in cell culture in several signaling pathways.

Signaling pathway	Treatment time	Concentration	Cells	Results	Ref
Akt-GSK3*β*	9 h	50 *μ*M	Human osteoblastic cell line (Saos-2)	(i) Curcumin was cytoprotective since it significantly improved the viability of cells exposed to H_2_O_2_ and reduced H_2_O_2_-induced apoptosis. (ii) Curcumin preserved the potential of mitochondrial redox, reduced the mitochondrial oxidative status, and improved the mitochondrial membrane potential and functions. (iii) Curcumin increased of phosphorylated glycogen synthase kinase-3*β* (GSK3*β*) and phosphorylated protein kinase B (Akt) levels.	[22]
Wnt/TCF and Wnt/*β*-catenin	24 h	50 or 100 *μ*M	Human aMSCs	(i) Curcumin protects from cell death caused by H_2_O_2_. (ii) Curcumin increased the osteoblast differentiation that is inhibited by H_2_O_2_. (iii) Curcumin attenuated the oxidative stress and the inhibition of Wnt/*β*-catenin signaling. (iv) Curcumin can indorse osteoblast differentiation and protect the inhibitory effect elicited by oxidative injury.	[23]
PI3K/AKT/Nrf2	7 days	0.1 *μ*M	Periodontal ligament stem cells	(i) A suitable concentration of curcumin had no cytotoxicity and could indorse osteogenic differentiation. (ii) Curcumin can stimulate the osteogenesis, and the influence is related to the PI3K/AKT/Nrf2 signaling pathway.	[24]
ER stress	1, 3, 7, or 15 days	10 *μ*M	C3H10T1/2 mesenchymal cells	(i) Curcumin exhibited no cytotoxic activity at concentrations up to 10 *μ*M. Curcumin-induced mild ER stress increases differentiation of osteoblast by ATF6 expression in C3H10T1/2 cells.	[20]
GSK3*β*-Nrf2	7 days	0.25 *μ*M	MC3T3-E1 cells	(i) GSK3*β*-Nrf2 activation and ROS scavenging can be the key responsible mechanism for prosurvival and differentiation-stimulating actions of curcumin in H_2_O_2_-induced oxidative damage of MC3T3-E1 cells.	[25]

## Data Availability

The raw/processed data required to reproduce these findings can be shared at this time.
